# Evaluation of moral case deliberation at the Dutch Health Care Inspectorate: a pilot study

**DOI:** 10.1186/s12910-016-0114-4

**Published:** 2016-05-21

**Authors:** Wike Seekles, Guy Widdershoven, Paul Robben, Gonny van Dalfsen, Bert Molewijk

**Affiliations:** Medical Humanities, VU University Medical Centre, Amsterdam, The Netherlands; The Health Care Inspectorate (IGZ), Utrecht, The Netherlands; University of Humanistic Studies, Utrecht, The Netherlands; Institute of Health Policy and Management (iBMG), Erasmus University Rotterdam, Rotterdam, The Netherlands; Centre of Medical Ethics, HELSAM, University of Oslo, Oslo, Norway

**Keywords:** Moral case deliberation, Ethics support, Evaluation, Health care inspectorate, Health care regulation

## Abstract

**Background:**

Moral case deliberation (MCD) as a form of clinical ethics support is usually implemented in health care institutions and educational programs. While there is no previous research on the use of clinical ethics support on the level of health care regulation, employees of regulatory bodies are regularly confronted with moral challenges. This pilot study describes and evaluates the use of MCD at the Dutch Health Care Inspectorate (IGZ).

The objective of this pilot study is to investigate: 1) the current way of dealing with moral issues at the IGZ; 2) experience with and evaluation of MCD as clinical ethics support, and 3) future preferences and (perceived) needs regarding clinical ethics support for dealing with moral questions at the IGZ.

**Methods:**

We performed an explorative pilot study. The research questions were assessed by means of: 1) interviews with MCD participants during four focus groups; and 2) interviews with six key stakeholders at the IGZ. De qualitative data is illustrated by data from questionnaires on MCD outcomes, perspective taking and MCD evaluation.

**Results:**

Professionals do not always recognize moral issues. Employees report a need for regular and structured moral support in health care regulation. The MCD meetings are evaluated positively. The most important outcomes of MCD are feeling secure and learning from others. Additional support is needed to successfully implement MCD at the Inspectorate.

**Conclusion:**

We conclude that the respondents perceive moral case deliberation as a useful form of clinical ethics support for dealing with moral questions and issues in health care regulation.

## Background

Quality of health care is an important issue in health care policy. In the Netherlands, the Dutch Healthcare Inspectorate (IGZ) is the body appointed by the government to supervise and regulate the quality of healthcare. It is an independent part of the Ministry of Health, Welfare and Sports (VWS). Its position and role is comparable to the English Care Quality Commission (CQC). The IGZ enforces 25 laws. It is authorised to use the following regulation and enforcement instruments in order to do so: advice and incentives, corrective measures, administrative measures and measures under criminal or disciplinary law. The regulation of the IGZ is based on the theory of ‘responsive regulation’ of Ayres and Braithwaite [1]. Parties being regulated are considered to be trustworthy and intrinsically motivated to deliver good and safe care. Strategies of regulation should be flexible, in synergy with the context of those being regulated. Regulatory compliance is encouraged by using cooperation, persuasion, inspection and enforcement notices in the first instance, and secondly by applying heavier measures in the case of riskier behaviour. This vision is often described as ‘high trust, high penalty’. This vision, and regulating in general, raises various moral questions. These moral questions concern both concrete cases in which the IGZ inspector has to decide to respond to a health care institutions and more general moral questions concerning professional and organizational identity. For example questions such as: What is good regulation? When is regulation applied in a correct manner? What is a good inspector? At another level, these moral questions lead to a meta-question: What is a good way of dealing with these moral dilemmas and normative questions regarding regulation?

Moral issues are inherent to the work of care givers. Is it, for example, the case that a care giver should inform the partner of a patient who has a sexually transmitted disease? The work of the Inspectorate does not directly influence patient care. Yet, inspectors can encounter moral dilemmas too. They may, for instance, doubt whether they should inform a healthcare institution about a procedure against a professional [see example 1].

*“Several charges of sexual intimidation by a professional are being investigated. He is temporarily relieved of his duties and subsequently fired. Coincidentally, I heard that this professional has applied for a position at another health care organisation”. The moral question of the inspector was: “Should I warn this organisation?”* – Health Care Inspector, 2014

**Example 1.** Example of a moral case in health care regulation

It is often assumed that attention for ethics in general, and systematically and methodically reflecting upon moral issues in particular, can contribute (in)directly to both the quality of care and that of the professional [2, 3]. In addition, to a substantive focus (What is the good and right thing to do?), moral reflection has a procedural and analytical focus: How do we reason? What are our judgments and decision-making processes based on? What, apart from the content, is a good judgment?

Reflection on moral issues is of great importance for both the quality of the work of the IGZ and the publics’ trust in health care in general since the Dutch Inspectorate operates continuously within a public and political sphere [4]. A recent report of the Dutch Scientific Council for Government Policy (WRR) [5] on supervising public interests states the contrary demands that supervisory bodies have to deal with; less supervision on the one hand and more supervision on the other (during incidents or cases with media attention). The report describes this as a paradox: *“the struggle to limit supervision in a sector in incident-free periods (give the sector more responsibility, cut down on bureaucracy and expense) versus the tendency to increase it following incidents (expand and intensify supervision, make it stricter)”*. The report also describes what changes are needed in supervisory policy and practice to make government supervision more valuable to society. It entails seven recommendations to the Dutch government. Despite the recommendations of the Ministry of VWS to increase the attention for the role of ethics in care regulation [6], the WRR recommends no such thing in their report. The role of moral reflection in regulation has not yet been explored, not in daily practice or in scientific research. As far as we know, this is the first scientific evaluation of moral reflection in governmental regulation.

A method to discuss moral questions in health care is moral case deliberation (MCD). MCD is a systematic dialogue in which participants reflect upon their moral question, their presuppositions and their way of reasoning on the basis of a concrete experience. The moral question of a concrete case of the professional is examined by means of a dialogue. The aim is not primarily to determine who is right, but to understand how and why the other person thinks about the moral question in a certain way [7]. MCDs are led by a trained facilitator [8–11]. Moral questions focus on *What should we consider as the morally right thing to do in this specific situation and how should we handle it in the right way?* In general, MCD has three, co-existing, goals [8]. The first goal is to reflect on a case from daily practice and to improve the quality of care within that specific case. The second goal is to reflect on what it means to be a good professional and tot enhance the professional’s moral competencies. This refers to a set of skills and abilities that enhance moral behaviour, for example to be aware of one’s own personal and professional values and the ability to identify moral aspects of one’s profession. The third goal of MCD is to improve care on an organizational level through reflection on institutional or organizational issues, like recurrent themes or policy-making [12, 13]. MCD has its origin in traditions of pragmatic hermeneutics and dialogical ethics [2, 14]. These approaches emphasize the importance of processes of meaning-making and moral judgement in relation to concrete practical experiences [15]. In MCD, participants reflect upon a moral question in a specific practical experience. This practical approach to ethics has proven to be useful for professionals in various health care organisations [8, 12, 16–18]. We expect moral reflection to be useful for the IGZ because the chief questions posed in moral philosophy are ‘What kind of person should I be?’, and, second, ‘What kind of actions should I undertake? Health care inspectors are confronted daily with these two core questions when regulating the quality of care, in which they have to relate to the formal rules, the IGZ policy, the political dimension, the media and their own professional view on good regulation. Although the topic shifts from good care to good regulation, MCD may still be relevant, as individuals have to reflect on what is good and how to do good in a professional setting. Likewise, organizational ethical cases fall also regularly under the topic of a health care MCD (e.g. priority setting).

In this pilot study, MCD is introduced in the context of the Dutch Health Care Inspectorate in order to support employees in dealing with moral questions in their professions. The research questions of this pilot are threefold: 1) What is the current way of dealing with moral issues at the IGZ?; 2) What are the experiences with and evaluation of MCD?; and 3) What are future preferences and (perceived) needs regarding dealing with moral questions at the IGZ?

## Methods

### Design

This research is an explorative qualitative pilot study, illustrated with data from questionnaires. We included employees involved in the primary process of inspection (senior inspectors and reporting centre employees) based on voluntary registration. Together, these participants formed one MCD-group, which met eight times for a MCD. All participants gave informed consent for participation in the study.

### Recruitment

Participants for the MCD-group were recruited through the intranet of the IGZ. Eligible participants could volunteer to participate in the MCD-project. All 18 registered eligible participants were invited to an information meeting. Additional information on MCD and the pilot study in specific was given during this meeting. After this meeting, a number of participants were excluded due to lack of availability during the scheduled MCDs. In total 10 participants were included for the pilot study; one of them stopped participating during the pilot for external reasons. We included employees from different programs of the IGZ (see Research sample in Results section) in order to keep the research sample as representative as possible. We did not specify other specific inclusion criteria. For the interviews, we also included 6 employees of IGZ, so-called representative key stakeholders, with a broad view on the IGZ organization. These stakeholders (including program directors, chief inspectors and the inspector-general) were interviewed by means of a semi-structured protocol. These employees were chosen because of their knowledge of and view on the primary process of IGZ, given their function and work experience.

### Moral case deliberation

In this pilot study, eight MCDs were organized in the period of December 2012 to July 2013 (approximately one meeting per month). Each of these MCDs was scheduled for 90 min at the headquarters of the IGZ. One week before the start of a MCD, all participants were asked to send out their own case in which they personally experienced a moral challenge. During the meeting, participants chose one of these cases to discuss in the MCD. Authors BM and GvD alternately were the MCD facilitators. Within this pilot study we used the dilemma method for MCD [19]. The dilemma method consists of 10 steps (introduction, presenting the case, formulation of the dilemma, thoroughly examining the situation through questions for elucidation, analyzing values and norms, formulating alternative actions, making individual well-founded judgments, comparing judgments in dialogue, conclusion, and evaluation). All MCD sessions were audio taped but not transcribed. Based on the audiotapes, a confidential report was made after each MCD. This was distributed among all MCD participants for a final member-check. Incidentally, reports were slightly adjusted for privacy reasons.

### Evaluation of MCD

**Fig. 1:**
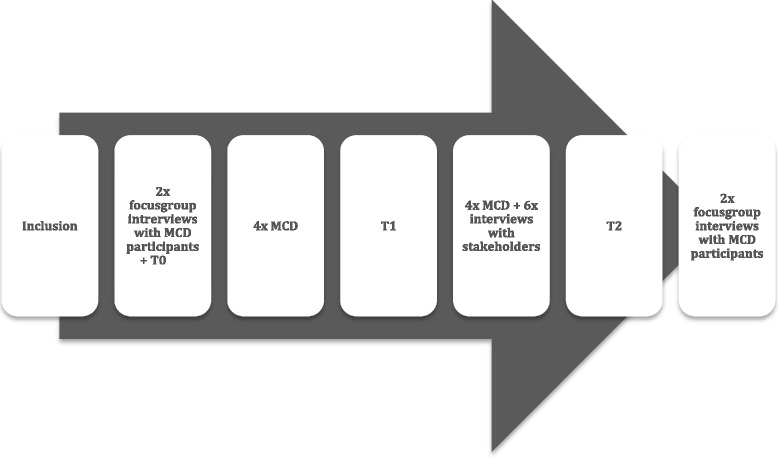
Chronological order of data collection

During this pilot study, several methods were used to collect data (see Fig. 1). Prior to the MCDs, two focus groups were organised with the future participants (2x5 participants). During these focus groups several topics were discussed: which moral issues arise in health care regulation, how do they deal with these issues today and what the expectations are regarding the MCDs. Participants filled in questionnaires after the focus groups and prior to the MCDs (baseline; T0), during the MCDs (after 3 months; T1) and afterwards (after 7 months; T2). These questionnaires assess outcomes of MCD and perspective taking (see section Questionnaires). Every MCD was also evaluated *after* every meeting via a short evaluation questionnaire. After the last MCD, again, two focus groups were organised. During these focus groups the MCDs were evaluated, the insights regarding good regulation were discussed, the effects and outcomes of MCD were inventoried and the future of ethics support at the IGZ were discussed.

### Questionnaires

Participants filled in questionnaires to illustrate the qualitative data.

#### Outcomes of MCD

The possible outcomes of the MCDs were assessed with the EURO-MCD [3]. This questionnaire consists of 26 items, which reflect possible outcomes of MCD. In the baseline measurement the participants are asked to assess the importance of each outcome (on a 5-point Likert Scale: very important – not important/cannot take stand). Examples of items are: *I see ethically difficult situations from different perspectives* and *Enables me and my co-workers to decide on concrete actions in order to manage the ethically difficult situations.* The questionnaires for the intermediate (T1) and final (T2) measurement have an extension: in addition to the importance of outcomes, participants are asked to indicate whether they have experienced the outcomes during the MCD sessions and in daily practice (on a 5-point Likert Scale: in high degree – not reached/cannot take stand). The EURO-MCD is currently used in several European countries [20].

#### Evaluation of MCD

After each meeting, a short questionnaire was distributed on the quality of the MCD and the facilitator, the relevance of the case and the degree in which the participants learned from the MCD. This questionnaire was used 7 times in total (from the 2^nd^ until the 8^th^ MCD).

#### Perspective taking

Perspective taking was assessed with the Perspective Taking Scale [21]. This scale is based on the questionnaire of Davis [22]. The 6 items are statements, for example: *Before criticizing somebody, I try to imagine how I would feel if I were in their place*. The participant rates the statement on a 5-point Likert-scale (strongly agree – strongly disagree). Cronbach’s α ranged between 0.68 for the pre-test and 0.61 for the post-test. For this study we translated the questionnaire into Dutch.

### Analysis

The research questions are answered by qualitative data and illustrated with examples from the quantitative data.

### Qualitative analysis

All interviews (pre- and post-test focus groups and interviews with stakeholders) were fully transcribed. The data was analysed based on the core questions of the interviews. Initial analysing was performed in line with quality criteria described in the literature, remaining open, staying close to the data and keeping codes simple and precise [23–25]. Two researchers (WS and BM) independently constructed short summary descriptions in tables, compared data, and involved research team members in the interpretation. A third researcher (GW) did a final independent check on data analysis. We discussed differences in interpretation among WS, BM and GW. After reaching consensus, we went back to the transcriptions in order to check our summaries. These summaries were sent to co-researchers PR and GvD for an additional check. After this process the interpretations were sent to the participants for a final member-check.

### Quantitative analysis

We calculated means and standard deviations of the short MCD evaluation and the perspective taking scale. The pre- (T0), intermediate- (T1) and post-test (T2) scores of the EURO-MCD are presented in actual number of participants.

## Results

The results section is divided into three topics that correspond to the central research questions of this pilot study. The results of the various research methods are combined for each topic. The three topics are: 1) current way of dealing with moral issues at the IGZ; 2) experiences with and evaluation of MCD as clinical ethics support, and 3) future preferences and (perceived) needs regarding clinical ethics support for dealing with moral questions at the IGZ.

### Research sample

The MCD group consisted of eight women and one man. These participants work in the primary process of health care regulation (senior inspectors and reporting centre employees). The participants differed in professional background. The programs of the IGZ that were represented were: hospital care, primary care, mental health care, elderly care, health care for people with disabilities and pharmacological care. The research sample does not fully represent the targeted population in terms of gender (of the (senior) inspectors 43 % is male and 57 % female). The small sample of respondents (*n* = 9) and its variations should be taken into account in the interpretation of the results. The conclusions of this paper are drawn on the qualitative data. When reading the results, it is important to bear in mind that these are based on a small and unrepresentative group of employees.

### Cases

Before every MCD, all participants sent in a case with a moral question or issue that they have or had experienced in their work as an inspector. The cases concerned various topics. These can be put under the following categories: 1) How should we relate to others?; 2) How should we cooperate with each other within the IGZ? 3) Intensified supervision or equity? 4) Is it allowed to give substantive judgements on care/profession?; 5) What is an appropriate role of the inspector (IGZ) in a conflict between other parties?; 6) What are the boundaries of the professional responsibility?; 7) Should we always adjust to the new and stricter IGZ policy? and 8) If and when do we have to share information?

#### How do employees currently deal with moral questions at IGZ?

In the focus groups prior to the MCDs, participants discussed how employees at IGZ deal with moral questions in their daily practice. According to all participants, there are no specific guidelines, codes of practice or policy agreements at the IGZ regarding specific moral themes or moral dilemmas. Furthermore, there is no specific suggestion or vision in how to deal with moral themes or moral dilemmas in general. One of the respondent’s says: “*And if there are any forms of support, they are not active enough*”. There are several forms over implicit and explicit professional consultation at the IGZ, but most of the time they focus on the content of the case and not specific on underlying values, norms and principles. The participants think this is an unfortunate situation, because*”it can be very useful to encourage others to start thinking about something as well”.*

The participants believe that they do not (sufficiently) learn from each other’s vision and methods of reflection to enhance better understanding and practice of good regulation. Most participants experience very few possibilities to discuss moral questions and moral issues in the organisation. Most participants agree that this is unfortunate, especially when you encounter a morally difficult situation in practice, where it is often difficult to determine your position as a professional. One of the participants formulated this as: *“exactly there were things go wrong, the help of IGZ is being invoked”*.

Like the MCD participants, the stakeholders interviewed conclude that there are no moral committees or guidelines. Sometimes moral or moral issues are discussed in a regular meeting when cases are discussed, but this also depends on the program director. Some of the stakeholders hope that their employees will come to them, but they are not convinced that they will actually do so. *"I hope my employees come to me, I have the impression that they do, but you never know".* However, the stakeholders agree that the program director is the designated person for the inspectors to discuss their moral issues, *“however this will depend on the relationship with your program director”*. Most stakeholders think that employees often consult each other. The stakeholders are convinced that there is a need amongst employees to discuss moral issues of regulation, but that there also will be a group that will, initially, not recognize the importance of such discussions. One last theme mentioned by the stakeholders is the recognition of moral aspects of regulation. *“I guess not everyone is aware of the fact that something is an moral dilemma. They are considered to be difficult situations, however by asking it turns that something is an moral dilemma. It is not very often labelled as an moral dilemma”*. The stakeholders consider this as an important first step in learning to deal with moral problems: learning to recognize what a moral dilemma is.

### Summary

There are no specific guidelines or vision on how to deal with moral issues at the IGZ. Furthermore, issues are not always recognised, as moral issues and learning what a moral issue is would be a first step for employees of the Inspectorate.

#### Experiences with and evaluation of moral case deliberation

*Evaluation of the MCDs.* The MCDs are positively evaluated by the 9 participants. In total they filled in 56 evaluation questionnaires after the meetings. The quality of the facilitator was rated with an average of 8.00 on a 1 to 10 scale (*sd* = .71) and the quality of the MCDs with an average of 8.01 (*sd* = .50).

The moral questions that were discussed during the MCDs were representative of the moral issues in regulation (*m* = 4.43 on a 1 to 5 Likert scale; *sd* = .33). Accordingly, the participants thought that the MCDs were useful (*m =* 4.41; *sd* = .17) and instructive (*m* = 4.42; *sd* = .10) for daily practice. During the MCDs the participants allowed each other to finish their reasoning (*m* = 4.27; *sd* = .22) and they experienced that their input was taken into consideration by the other participants in the final decisive round (*m* = 4.29; *sd* = .19).

Both the questionnaires and the evaluation interviews revealed that the participants were very positive about MCD. During the evaluation interviews, the MCD participants used the following keywords to describe MCD: essential, meaningful (for oneself and for the IGZ), broadening one’s vision, open, program transcending, feeling secure, involvement, collegiality, trust and collectivity.

Participants state that they experienced the MCDs as rewarding and considered it important to reflect *together* on moral questions and moral dilemmas that they regularly encounter in regulation. During the MCDs, the participants became more aware of the different perspectives present in a case, and of the possibility to learn from other perspectives. One participant states that it is no longer *“just because we do it like this”*, but that he/she, as a result of MCD, is doing more research (i.e. gathering more facts and discussing with colleagues) before making a decision. Another participant illustrates that MCD is *“taking a step back”*; “*My first thoughts are not always the right ones”.* Participants learned to listen more carefully to others and adjust their own initial thoughts. A valuable aspect of MCD is that it transcendents various disciplines of IGZ. Participants are confronted with different perspectives on a case and, additionally, gain more knowledge regarding the methods and procedures of other disciplines of the IGZ. This leads, according to the participants, to a better understanding of each other’s work.

During the MCDs, the dilemma method was used to facilitate the dialogue. Most participants thought it was a good method; however a few participants criticized some aspects of this method. These participants expressed the view that the dilemma method focused too much on one individual in the group. Furthermore, the participants regretted that most cases were not discussed (only 1 out of 9) in a MCD because they all contained important and urgent themes from daily regulation. After a few MCDs it turned out that some participants experienced some difficulties with formulating their moral question concerning their case. There was some confusion about when a question is a moral question (and when not). Furthermore, some participants lacked time to find an appropriate case for the next meeting. They conclude that the presented cases are a representative mix of organizational dilemmas and dilemmas regarding health care regulation.

*Outcomes of MCD.* The possible outcomes of MCD were assessed by the EURO-MCD [3].

##### Importance of MCD outcomes

Table 1 shows the outcomes of MCD that were considered to be most important by the participants. The number indicates how many respondents rated the outcomes *important* or *very important* on a 5-point Likert Scale (see Questionnaires). The table contains all outcomes that are rated important or very important by all participants on post-test. The deviant scores on the intermediate test could not be explained due to the small sample size.

**Table 1:** Outcomes of MCD considered important in number (*n* = 9) of participants

Outcomes MCD	T0 (baseline)	T1 (intermediate)	T2 (post)
I listen more seriously to others’ opinions	6	7	9
Develops my skills to analyse ethically difficult situations	8	7	9
Find more courses of actions or order to manage the ethically difficult situation	8	8	9
Gives me more courage to express my ethical standpoint	6	6	9
Better mutual understanding of each other’s reasoning and acting	9	6	9
I and my co-workers become more aware of recurring ethical situations	8	6	9
I see the ethically difficult situations from different perspectives	9	8	9
Increases awareness of my own emotions regarding ethically difficult situations	6	8	9
Develops my ability to identify the core ethical question in the difficult situations	8	7	9

As shown in Table 1, the participants perceive *seeing ethically difficult situation from different perspectives* as important. In addition, the data of the Perspective Taking Scale [21] show that the score on the item *I try to look at everybody’s side of a disagreement before I make a decision* increased largely over time and is rated highest of all items of the questionnaire (T0: *m =* 2.87 and *sd =* 0:35, T2: *m* = 3.44 and *sd* = 0:53). This might indicate that participating in MCD contributes to (the learning of) taking different perspectives on moral issues or moral situations.

Two possible outcomes of the EURO-MCD were rated less important over time: *Consensus is gained amongst co-workers in how to manage the ethically difficult situation* and *I and my co-workers examine more critically the existing practice/policies in the workplace/organization*. Respectively, T0: *n* = 9, T1: *n* = 7 and T2: *n =* 7 and T0: *n* = 8, T1: *n* = 6 and T2: *n* = 6.

### Experienced outcomes in daily practice

The intermediate- and post-test of the EURO-MCD contains an additional questionnaire in which respondents could rate to what extend the possible outcomes of MCD were actually experienced in daily work. Half of the 26 outcomes increased in daily practice over time. The three outcomes that were experienced the most (*in high degree or in rather high degree*) in daily practice are: *Consensus is gained amongst co-workers in how to manage the ethically difficult situation* (T1: *n =* 5 and T2: *n = 6*), *I and my co-workers become more aware of recurring ethical situations* (T1: *n =* 3 and T2: *n =* 8) and *Enhances possibilities to share difficult emotions and thoughts with co-workers* (T1: *n =* 4 and T2: *n =* 8).

Outcomes that were least experienced in daily practice were: *I feel more secure to express doubts or uncertainty regarding ethically difficult situations* (T1: *n =* 5 and T2: *n =* 3), *Increases awareness of my own emotions regarding ethically difficult situations* (T1: *n =* 2 and T2: *n =* 1), *I see the ethically difficult situations from different perspectives* (T1: *n =* 1 and T2: *n =* 3) and *I became more aware of my preconceived notions* (T1: *n =* 3 and T2: *n =* 1). Two of these outcomes were considered as the most important of MCD.

### Possible improvement of MCDs

A number of aspects of the MCDs need some improvement according to the participants. One important aspect is time management; the participants experienced structurally insufficient time for all steps of the method. The participants recommend taking more time for a MCD to give sufficient attention to the case under discussion. A final improvement would be to use various methods of MCD because the dilemma method was sometimes too focused on an individual instead of the IGZ or a team.

However, the participants indicated that they intended to change professional behaviour with respect to dealing with moral or moral issues in regulation. The intended changes consisted of:

- discussing more (and sooner)
- determining values, norms, arguments and subjectivity
- varying and changing perspective
- reflecting, creating time and space
- determining focus and aim in a certain situation
- systematically collecting information (facts)/weigh information
- staying close to oneself/ensure ones own integrity

### Summary

Participants evaluated MCD positively and find it a useful method for their daily work. Participants have learned form others, learned to see moral issues from different perspectives and, accordingly, changed their professional behaviour (reflecting, determining focus and discussing more with colleagues).

#### Future needs in dealing with moral questions at the IGZ

Both participants and stakeholders were asked how the IGZ should (ideally) deal with moral questions and moral issues in the future. First the opinions and ideas of the participants are described, followed by those of the stakeholders.

##### MCD participants

*Necessity.* All participants consider it important that the IGZ organizes some form of ethics support. According to the participants, ethics is *“an integral part of our profession”*. Ethics are connected to the professionalism and needed to optimize adequate regulation. However, it is important to keep ethics support close to the daily practice of regulation and related to casuistry.

*Security.* Several participants state that they think it is important to examine their views or ideas on a certain case. Yet, all participants experience little space to share moral dilemmas outside the MCD sessions while they at the same time have a need to do so: *“If you want to reflect on something, then you [need] people you trust”.* The regular meetings within the organisation should be safer to discuss moral questions, and MCD can help in this respects. *“It [MCD] is a useful method to enhance trust, since this is lacking”.*

*Learning from others.* Currently, employees experience cultural differences between the disciplines within IGZ. In response to the possible role of MCD a participant says: *“I consider the inter-disciplinary transcendence [at the IGZ] as really important”*. For better mutual understanding it appears to be of importance to deploy ethics support broadly in the organisation, *including* the management.

*Implementation*. A number of participants indicate that it is necessary to invest time to motivate people for ethics support or MCD at the IGZ. *“We need some individuals who will take the initiative”*. Some of the participants say ethics should be firmly embedded in the organization. One suggestion is to integrate methodical dealing with moral questions and substantive reflections on good regulation in the inspectors’ education program.

##### Stakeholders

*Awareness.* According to all stakeholders it is important that employees of the IGZ learn to recognize moral questions and dilemmas that are associated with de work field. A first step would be creating awareness trough education (for example in the internal training institute; the Academy), a course applied ethics specific for IGZ or presenting moral questions to employees.

*Necessity.* Most stakeholders agree that it is important to offer some form of moral support to deal with moral questions at IGZ. One of the stakeholders says: *“IGZ employees need it and it is really needed in the organisation”.* Moral dilemmas are inherent to regulation and they are intertwined with the work of the IGZ. Therefore it is not desirable to organise separate meetings or specific guidelines for ethics support. Almost all stakeholders agree that (a way of) dealing with moral questions and moral dilemmas should be integrated in the current organisational structure. Ethics support should stay close to the core of the work. Moral dilemmas should be discussed with reference to examples and preferably *“the cases should come from the employees themselves”*.

*Security.* The stakeholders think it is important to create an opportunity to discuss moral issues with colleagues. One of the stakeholders suggests discussing moral issues in a peer-group. However, in order to enter in a dialogue with each other, it is important that the internal culture of the IGZ is sufficiently secure. The creation of a secure environment still needs attention. *“You have to expose yourself and that requires a secure environment. You are going to be vulnerable”.*

*Role of the managers*. A number of stakeholders suggest making the MCD-participants ambassadors for discussing moral issues in regulation. Furthermore, the stakeholders believe that managers have to set an example in doing so. *“Change of cultural environment will occur at its best when managers set an example”.* One of the stakeholders proposes the opportunity to discuss moral issues systematically in the management as well. *“Personally, I think a kind of ethics committee for the whole organisation would be good. That we also become aware of the moral issues that occur at the level of the management”.*

*Support.* To create the opportunity and security to discuss moral issues in the organisation, support is needed among the inspectors. The stakeholders do not entirely agree to what extend the inspectors feel the need to discuss moral issues. One stakeholder says: *“I expect that the majority feels a certain turmoil and needs to do something with this”.* While another stakeholder mentions: *“There will be a large number of people who think this is absolute nonsense”.* The stakeholders that are less positive about the needed support amongst the employees suggest discussing moral issues within the various programs before it is expanded in the organisation.

### Summary

According to the respondents the IGZ needs ethical support to deal with moral issues in health care regulation. This should be firmly imbedded in the organisation. Moral reflection should be offered in a secure environment and create awareness of moral issues in regulation.

## Discussion

This pilot study shows that the respondents perceive moral case deliberation as a useful form of clinical ethics support for dealing with moral questions and issues in health care regulation. Both participants and stakeholders express a need for ethics support. The results show that MCD is perceived to be rewarding, meaningful and program transcending. Participants learned to view things from different perspectives, learned from others and changed their behaviour in daily practice (e.g. reflecting and determining aim and focus in a certain situation). Furthermore, the three co-existing goals of MCD [12, 13] are emerging well (partly realised) from the data. In order to be useful, it is important that ethics support is verified and supported by stakeholders from the organisation. The data confirms that this is true for the health care inspection.

### Need for ethics support

The experienced need for structural moral support in regulation of health care we found is in line with studies conducted in health care institutions. Slowther and colleagues [26] state that in the UK, of the respondents (health care professionals) who perceive a need for a clinical ethics support service that 89 % agreed or agreed strongly that their National Health Service trust should have such a service. Accordingly, in a Dutch study (*n* = 2137; response rate 56 %) board members and ethics support-staff of health care institutions express a need for ethics support [27]. The need for ethics support in regulation can be explained by the moral questions that arise out of the interaction between health care and health care regulation and between the different views on quality of care and how to effectuate an improvement of the quality of care. In health care regulation, professionals are affected by moral stress and concern due to the continuous confrontation with (structural and/or serious) problems in health care institutions. In turn, the regulation itself aims at improving the quality of care, thereby implicitly and explicitly defining the quality of health care and its limits (i.e. which quality is good enough?).

### Learning from others

According to the participants, MCD gives more insight in how colleagues from other disciplines work and think. Participants perceive “*a better mutual understanding of each other’s perspectives”* and *“seeing an moral difficult situation from different perspectives”* as important outcomes of MCD. This finding supports previous studies [28, 29]. In both studies MCD was offered in interdisciplinary groups, apart from existing communication structures. Both studies state that participants experience *stimulation of broadening thinking* and *a sense of connecting*. Participants are confronted with different perspectives on a case and, additionally, gain more knowledge and insights regarding methods and procedures of other disciplines. MCD can create not only an opportunity to exchange ideas but also stimulate broad thinking from different perspectives. The item *“I try to look at everybody’s side of a disagreement before I make a decision”* on the perspective scale largely increased during and after the series of MCD. Despite the perceived importance, the outcome *seeing moral difficult situations from different perspectives* has until now, unfortunately not been experienced in daily practice of health care regulation. A possible explanation could be that it takes some time and experience in the thinking process of MCD before professionals can translate the moral competencies into their daily work.

MCD might contribute to a better interrater reliability of inspectors (or point out the lack of this reliability). A study by Tuijn and colleagues [30] shows that there is a large variation in the judgments of inspectors. Work towards a more consistent and better interrater reliability starts with the understanding of the large variation and the substantive underpinning of the desired consistency. MCD does not primarily aim to reduce variation between perspectives and judgements, but it can generate more insight into perceptions and reasoning of colleagues. Because both perceptions and reasoning are made explicit in a MCD this can lead to a better grip on the variation in judgements, which can lead to a reduction in the variation in the interrater reliability. Future research should study the relationship between moral support and effective regulation.

### Security

An important outcome of the MCD series, according to the participants, is that they experienced MCD as a safe and secure base for sharing moral dilemmas and moral uncertainties. As a consequence, trust and a secure organisational environment is important in order to be able to learn to deal with moral questions and concerns. The pilot study at the IGZ shows that security is a prerequisite for discussing moral issues. Both MCD participants and the interviewed stakeholders doubt whether there is currently enough security for sharing and discussing moral issues within the IGZ. If the organisation is perceived as relatively unsecure, this might lead to less discussion about good regulation. As shown in other organizations, MCD requires a relatively more open and more secure atmosphere and attitude than other forms of consultation [31]. The success of this MCD pilot indicates that a secure environment to discuss moral issues within the context and structure of MCD is possible and feasible within the IGZ. The participants state that they experienced trust and security to express themselves regarding their own moral challenges, both during the MCD meetings *and* in daily practice after participating in MCDs.

On a professional level, individuals often express insecurity in expressing their professional doubts and fears [8] because others can interpret those doubt and fears as signs of professional weakness. In relation to the above-mentioned collectivity or, as stated by a participant *togetherness*, MCD can contribute in creating a safe environment on both an organisational and personal level to discuss moral challenges. The study of Molewijk and colleagues [12] in a psychiatric hospital showed that participants in structural MCD-groups felt more secure in dealing with moral questions and dilemmas. In our current pilot study, the MCD-outcome “*Gives me more courage to express my moral standpoint”* is considered very important by the participants. On the contrary the outcome *I feel more secure to express doubts or uncertainty regarding moral difficult situations* was still rare in daily practice. It is recommended that future research on dealing with ethical challenges within an organisational context focuses on long-term effects of learned moral competencies in daily practice.

### A new context for MCD

As mentioned in the introduction, this has been a new context for the implementation of MCD. Although we did not explicitly compare this context with the regular health care institutions in which MCD is usually implemented, some differences can be mentioned. With respect to the content of the MCDs we observed that the cases often focused on organizational and political issues, since the inspectors have to balance between formal rules from the Inspection, the law, the political context and the role of the media. We also noticed a more formal reasoning and argumentation of the participants, probably due to the formal requirement that inspectors have to reason clearly why and how they response to health care institutions or persons who fail to deliver high quality care.

### Future support

Moral challenges are inherent to the profession of the inspectors. Given the need for ethics support and based on the experiences of the participants and the input of the stakeholders we suggest that the IGZ expands the experiences with MCD within the organisation. This could be thematic MCD series on a specific topic, inter-disciplinary MCD sessions or an ad hoc MCD session when a team encounters moral uncertainty or moral disagreement. A number of employees have to be trained as MCD facilitators in order to require internal MCD expertise. Another way of developing professionals’ moral competence could be reached through integrating basic knowledge and expertise on ethics (support) in the IGZ internal education program for IGZ inspectors. It is important to create a working or steering group who can coordinate and reflect upon the attention for ethics and ethics support within the IGZ.

### Strengths and limitations

A strength of this study is that it uses both quantitative and qualitative data in order to provide a complete overview on the research questions. Furthermore, we chose a natural setting to evaluate MCD. However, the presence of the researchers WS and BM during the MCDs and the role of BM as both researcher and MCD facilitator might have had some effect on the outcomes.

This pilot also has some limitations. The conclusions are based on data from a small sample size and restricted to the health care Inspectorate in the Netherlands. For this reason, the findings cannot be generalized and are not representative for health care regulation in general. The quantitative presented information is not generalizable; it is used as an illustration. A further limitation is that participants were invited to join the pilot study based on their own interest for moral case deliberation (sampling bias; non-random sample and self-selection bias). This probably has led to a more positive evaluation than it would have done when employees in general were invited to participate. We tried to minimize this bias by checking our findings with the supervisory group, during interviews, presentations on internal workshops and at a presentation for the management team of IGZ.

## Conclusions

Regulation is inherently normative. Working in the field of regulation comes with ethical challenges. Until now, there have been no previous studies on ethics support services in regulation. Evidence based instruments and protocols for regulation do not make the moral dimension of regulation disappear. This pilot study is the first step in evaluating this moral dimension of governmental supervision in general. Systematic attention for ethics support and moral reflection can contribute to the expertise and professionalism of the primary process in health care regulation. Based on this pilot, we conclude that moral case deliberation is perceived as a useful kind of clinical ethics support for dealing with moral issues in the Dutch health care inspectorate.

## Declerations

### Ethics

We did not seek the approval of a medical ethics committee. This study focuses on the evaluation of professional practice instead of medical science and therefore does not need, according to Dutch legislation, a separate approval from a medical ethics committee.

#### Consent to participate

Prior to the evaluation pilot an information meeting was organized to fully inform participants about the study. Informed consent to participate in the study was obtained from participants and a statement to this effect appears in the method section of the manuscript.

## Availability of data and materials

The survey instrument, and all aggregated data are reported in the manuscript. The database can be made available on request to the authors.

## Abbreviations

CQC: Care Quality Commission
IGZ: Dutch Health Care Inspectorate
MCD: moral case deliberation
VWS: Ministry of Health, Welfare and Sports
WRR: The Scientific Council for Government Policy
